# Hyperthermic Intraperitoneal Chemotherapy and Recirculation with CO_2_: A Safe Technique

**DOI:** 10.3390/jcm11206152

**Published:** 2022-10-19

**Authors:** Remedios Gómez-Sanz, Enrique Ovejero-Merino, Inmaculada Lasa-Unzúe, Adela López-García, Ruth Marcos-Hernández, Javier Mínguez-García, Francisca García-Moreno Nisa, Fernando Mendoza-Moreno, Manuel Díez-Alonso, Miguel A Ortega, Melchor Álvarez-Mon, Alberto Gutiérrez-Calvo, the Spanish PRS Collaborating Group

**Affiliations:** 1 Department of General and Digestive Surgery, Príncipe de Asturias Teaching Hospital, 28805 Madrid, Spain; 2 Spanish Group of Peritoneal Oncologic Surgery (GECOP), Principe de Asturias Teaching Hospital, University of Alcalá de Henares, 28001 Madrid, Spain; 3 Department of Medicine and Medical Specialities, Faculty of Medicine and Health Sciences, University of Alcalá, 28801 Alcalá de Henares, Spain; 4 Ramón y Cajal Institute of Sanitary Research (IRYCIS), Hospital Universitario Príncipe de Asturias, 28034 Madrid, Spain; 5 Immune System Diseases-Rheumatology and Internal Medicine Service, University Hospital Príncipe de Asturias, (CIBEREHD), 28806 Alcalá de Henares, Spain

**Keywords:** peritoneal carcinomatosis, intraperitoneal chemotherapy, cytoreductive surgery, intraoperative intraperitoneal chemotherapy, Spanish PRS Group

## Abstract

Introduction: Hyperthermic IntraPEritoneal Chemotherapy (HIPEC) has evolved as a treatment for peritoneal carcinomatosis in various tumors after a careful and complete cytoreductive surgery, and it demonstrated much better and longer survival than more traditional therapeutic schemas. Our objective has been to examine the safety, efficacy and survival achieved with closed technique with CO_2_-agitation system Combat PRS^®^ (Peritoneal Recirculation System: PRS). To achieve this, we compared the appearance of adverse events, mortality and survival with the described using classic techniques (open, closed without CO_2_-agitation) for the treatment of selected patients with peritoneal carcinomatosis; Materials and methods: We studied overall survival, disease-free survival and safety (morbidity and mortality) of the administration of HIPEC through a closed method technique with CO_2_ recirculation (Combat PRS^®^) in 482 patients from 11 Spanish hospitals; Results: The mortality of our technique (1.66%) was similar to other published techniques (open, closed). Morbidity exhibited a 9.96% rate of Clavien-Dindo (CD) III/IV complications in 482 patients, which was lower than in other series. Survival (overall survival (OS) and disease-free survival (DFS)) was similar to previously published results: 86% 1y-OS, 54% 3y-OS, 77% 1y-DFS and 31% 3y-DFS; Conclusion: The procedure with closed PRS with CO_2_ agitation is as safe as standard open and closed procedures for the administration of HIPEC after complete cytoreductive surgery, with similar and very low mortality (1.66%) and lower morbidity (9.96% CD III and IV in our series vs range of 20–40% in the majority of different series); only Kusamura had similar results, with 12% in 205 patients, using the closed technique without CO_2_ agitation).

## 1. Introduction

The combination of cytoreductive surgery and perioperative chemotherapy [1,2,3,4,5,6,7] is the treatment of choice for select patients with peritoneal carcinomatosis of many tumors (e.g., peritoneal pseudomyxoma, mesothelioma or colon tumors [8,9,10]), and it has great promise in other tumor types [11] (e.g., gastric [12,13,14,15] or ovarian origin [1,2,16]).

Cytoreductive surgery (CRS) aims to completely remove all macroscopically visible tumors, and perioperative chemotherapy (CT) acts in a complementarily way to eradicate microscopic residual implants [4,5,6]. Although cytoreductive surgery procedures have become quite standardized since the publication of peritonectomy techniques by Sugarbaker, this standardization has not occurred with intraperitoneal chemotherapy, which has many existing protocols involving different chemotherapeutic drugs, durations, temperatures and application methods. [17].

CRS with intraoperative chemotherapy are usually long and complex procedures, usually involving multivisceral and peritoneal resections, with great systemic surgical repercussion and the added toxicity of concomitant, intraoperative and chemotherapy, but the long-term results are encouraging [4,18,19].

The HIPEC rationale is deliver a higher dosage of chemotherapy on the locoregional extension of the tumor (the peritoneal surface) with lower systemic toxicity. The direct introduction of chemotherapy in the peritoneal cavity achieves this objective, but this is further improved by hyperthermia, which enhances the penetration depth of cytotoxic drugs. This depth is limited and, therefore, can be only effective in patients with minimal residual disease after complete CRS [20].

The drugs, methods of application and timing of chemotherapy, however differ between work groups, and new techniques and methods have evolved to optimize the application of chemotherapeutic agents. Although HIPEC is the most widely used procedure in leading oncological centers, it lacks uniformity [2,6,21], with extensive variability in chemotherapeutic drugs, chemotherapeutic contact durations and methods of administration. Open, closed, half-open techniques or treatment with peritoneal cavity expansion coexist with the most recent contributions of a laparoscopic method (PIPAC) and closed technique with CO_2_ agitation (Combat PRS^®^). The best technique remains controversial [22].

Theoretical advantages of a closed system with CO_2_ agitation (Combat PRS^®^) are to maintain a more constant temperature within the peritoneal cavity, to achieve a homogeneous distribution of the chemotherapy selected and diminish the risk for operating system, as its assembly is easy and staff have minimal contact with chemotherapy (only during final aspiration of the abdominal cavity); this has been tested in pigs [23]).

Our objective is to examine whether the closed technique with the CO_2_-agitation system (Combat PRS^®^) was a safe and effective treatment of select patients with peritoneal carcinomatosis in real world practice, in 11 hospitals in Spain.

## 2. Materials and Methods

**Figure 1:**
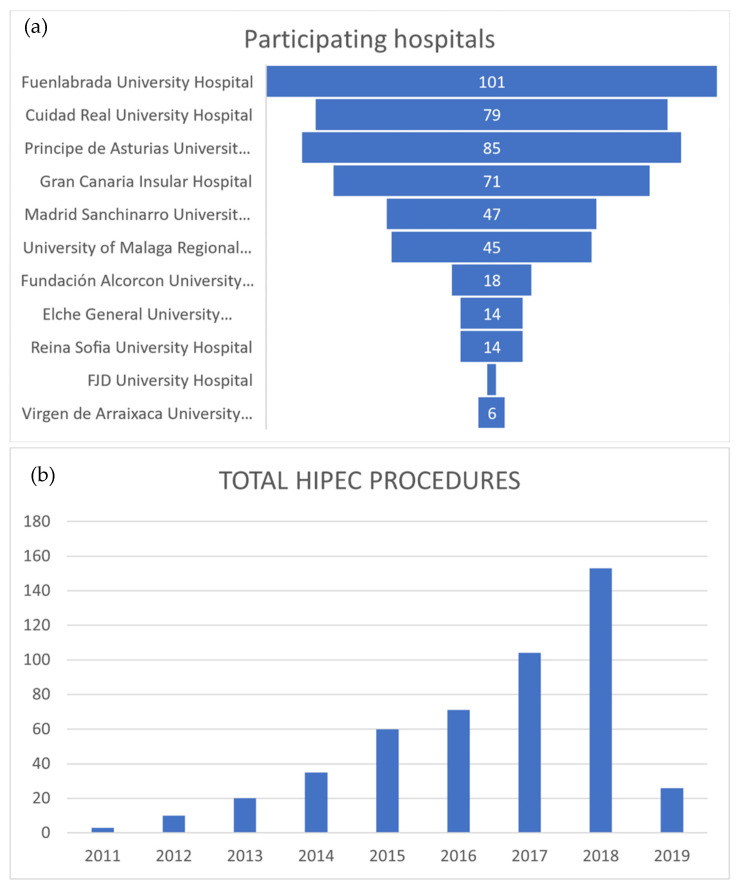

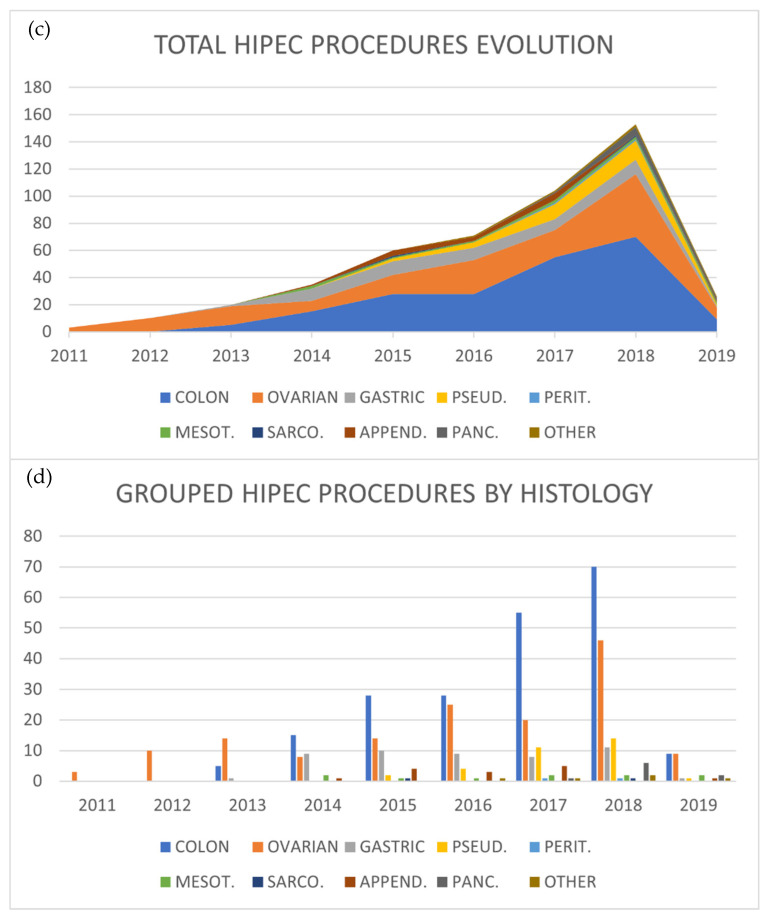
(**a**) Eleven participating hospitals. (**b**) Yearly increase of total HIPEC procedures. (**c**) Yearly increase of HIPEC procedures, with area proportional to histology, showing than the two main histologies are colon and ovarian origin. (**d**) Yearly evolution of HIPEC procedures with disaggregated yearly histology.

This study was a multi-center, retrospective study of 11 Spanish hospitals (Table 1, Figure 1a–d) that used the closed technique with CO_2_ agitation (Combat PRS^®^, Madrid, Spain) in the context of the multidisciplinary treatment of peritoneal carcinomatosis.

**Figure 2:**
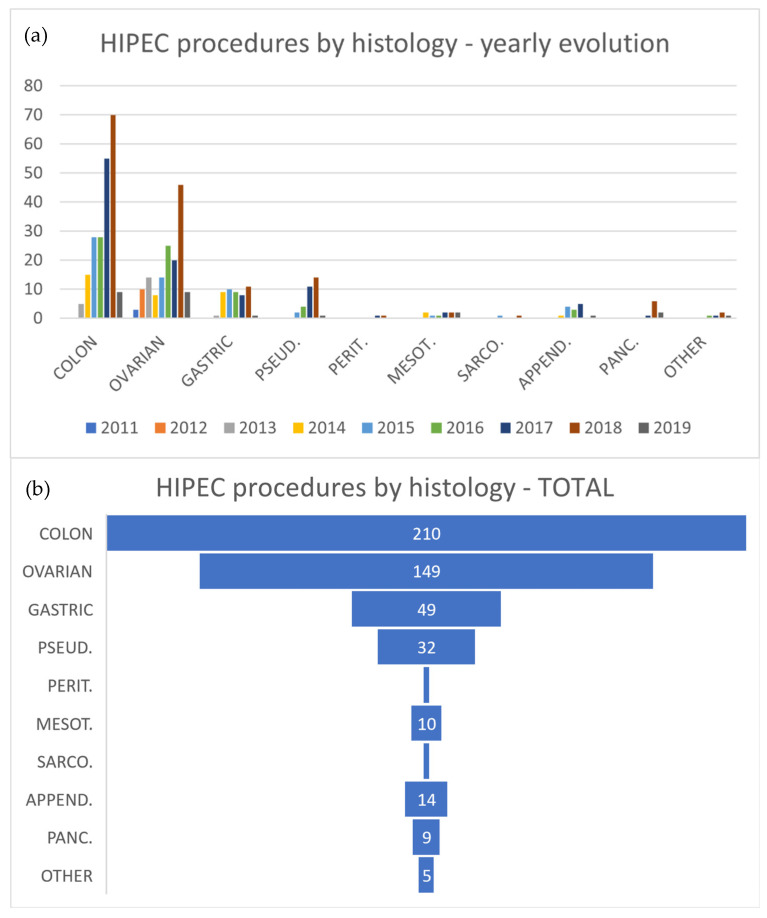
(**a**) Histology: yearly evolution of HIPEC procedures. (**b**) Total HIPEC procedures by histology.

The study period was from 2011 to February 2019, with a gradual and strong increase in the number of patients treated using this technique during this period (Table 2, Figure 2a,b).

The study included 482 patients who met the specific inclusion criteria (Table 3). All patients received HIPEC with a CO_2_-agitation system and the same perfusion machine (Combat PRS^®^). The surgical approach in every case was the one described by Sugarbaker [17]. The chemotherapeutics used and the treatment time varied according to the preferred protocol of each participating center.

Twenty-four variables were collected in a prospective database created for this purpose. The carcinomatosis index was quantified according to the peritoneal cancer index (PCI) described by Sugarbaker [25,26,27]. Data were collected on intraoperative complications related to the surgery, and data linked to HIPEC were collected separately. Complications detected in the postoperative period were recorded and codified according to the 2004 version of the Clavien-Dindo (CD) scale [28]. (Clavien-Dindo I and II are deviations from normal postoperative course solved pharmacologically; CD III are complications which require surgical/endoscopic or radiologic intervention without (IIIa) or with (IIIb) general anesthesia, and CD IV are life-threatening complications that require admission to Intensive Care Unit (ICU), with single organ (IVa) or multiorgan (IVb) disfunction. CD V is death: “mortality”). For the analysis of morbidity, CD III and IV have been taken into account (as reported in the main articles of Table 4). 

IBM-SPSS, version 22 (IBM, Armonk, NY, USA), was used for statistical analyses. Actual survival was calculated using Kaplan–Meier curves.

### Description of HIPEC Administration Technique

The closed technique with CO_2_ agitation is based on the existence of two closed circuits. One circuit is filled with chemotherapy agents, and the other circuit is filled with gas bubbles (CO_2_).

**Figure 3:**
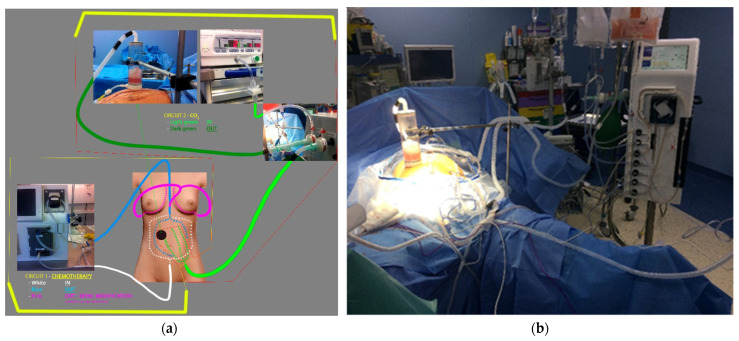
HIPEC and HITAC schematic view. Part (**a**) schematic view of the HIPEC system (white IN -blue OUT for chemotherapy, light green IN—dark green OUT for CO_2_; in pink, the HITAC modification, allowing chemotherapy recover from pleural cavities. Part (**b**) real intraoperative setting of HIPEC with Combat PRS® (Author: E. Ovejero-Merino).

After complete cytoreduction and exposure of all appropriate abdominal cavities, the base of the control device was passed through a small orifice (2 cm) in the abdominal wall to connect the cavity to a transparent extracorporeal cylinder (Figure 3a,b) that allowed us to monitor the proper level of filling and the intraabdominal pressure (which was approximately equal to the height of the water column over the skin level within this control device). This device was held in a vertical and stable position by an external arm tightly attached to the operating table. The three thinner multiperforated tubes for gas intake (Figure 3a (light green)) were positioned under the intestinal package and extended like a trident at the root of the mesentery. All tubes converged into a single tube, which exited the cavity through another small (1 cm) skin orifice over the left iliac fossa. This tube may be used to place a drain at the end of the procedure. A recirculation circuit of CO_2_ was established between these tubes (gas inlet) and the upper part of the control device (gas outlet (Figure 3a (black dot)).

Chemotherapeutic drugs in a liquid carrier solution were administered (inflow) via specially designed, multiperforated Y-shaped tubes with blunt ends (Figure 3a (white)), which were exteriorized through the lower part of the laparotomy and placed superficially over the visceral package. After entering the abdominal cavity, the solution was recovered (outflow) and recirculated through similar tubes with a larger diameter than the gas tubes (Figure 3a (blue)), which were exteriorized through the upper end of the laparotomy and positioned deeply in both parietocolic gutters. Once the tubes were placed, the laparotomy was closed as tightly as possible using continuous blocking stitches in the skin to allow impregnation of the abdominal wall with the chemotherapeutic agents during recirculation. After skin closure, recirculation of the solvent/carrier solution (transport liquid without chemotherapy) was started to test patency without external contamination risk. The solvent was generally the same liquid used for peritoneal dialysis (Physioneal 35, with 1.36% glucose) and preheated to 42 °C. After verification of correct recirculation, the gas was introduced to test the gas circuit. Once the desired amount of CO_2_ had been introduced, it only recirculated within its own circuit. Chemotherapeutic agents were added after confirmation that both circuits were functioning properly. Recirculation of CO_2_ aims to cause a turbulent flow that ensures a homogenous mixture of the chemotherapeutic agent solution and heat throughout the entire abdominal cavity.

The dose of chemotherapy was calculated according to the surface area of the patient’s body, and the amount of transport fluid depended on the capacity of each patient’s abdominal cavity, tissue compliance and degree of anaesthetic relaxation.

After completion of the recirculation time, the cavity was drained via the outlet tubes. Two full 5-min washes were performed with a clean, gas-free recirculation liquid to remove any remnant chemotherapeutic agents. After the last wash, the abdominal cavity was reopened, and any remaining liquid was manually suctioned. All disposable material was removed from the patient and directly placed into biological waste buckets to minimize the risk of contamination of operating room staff.

The diagram presents the variation used when it was necessary to open or resect any part of the diaphragm, which allowed cells to potentially reach the pleural cavity. This variation allowed perfusion and recovery of the recirculation fluid from the pleural cavity during the perfusion by connecting chest tubes to the outlet tubes. This variation was named HIperthermic ThoracoAbdominal Chemotherapy (HITAC).

## 3. Results

### 3.1. Description of the Series

Of the 482 patients, 66.4% were women and 33.6% were men. The average age at the time of the surgery was 59 years (CI ± 11.39).

In total, 210 cases were colon tumors, 149 cases were ovarian tumors, 49 cases were gastric tumors, 32 cases were pseudomyxoma, 14 cases were appendiceal tumors, 10 cases were mesothelioma, 2 cases were primary peritoneal tumors and 16 cases were other tumors (i.e., 9 pancreas, 1 endometrial, 2 sarcomas, 1 neuroendocrine and 3 GIST) (Table 2).

The global mean hospital stay was 13.4 days with 3.2 days in the ICU. There were no significant differences related to the type of tumor.

For the procedures performed in the cytoreduction, more than four procedures were performed in 215 patients (44.6%).

### 3.2. Peritoneal Carcinomatosis Index (PCI)

The clinical PCI was lower than the PCI during surgery in all the included tumors (Table 2).

### 3.3. Chemotherapeutic Drugs

For colon tumors, the most commonly used agents were mitomycin C for 60 to 90 min (46.2%) and oxaliplatin for 30 min (45.7%).

The preferred drugs for ovarian tumors were paclitaxel (61.7%) and the combination cisplatin/doxorubicin (16.1%) for 60 min.

For gastric carcinomatosis, the most frequent combination (42.9%) was cisplatin and mitomycin C for 60 min.

For pseudomyxoma tumors, mitomycin C for 60 min was used in 78.1% of the cases.

For mesothelioma tumors, most cases (66.6%) received the combination cisplatin and doxorubicin for 90 min.

### 3.4. Morbidity/Mortality

A total of 170 patients (35.27%) exhibited complications during their hospital stay, and we classified the adverse events using the Clavien-Dindo scale. Only 48 of these adverse events (9.96%) were serious (CD III/IV) (Table 5).

Variables, such as age, drug used, PCI, type of primary tumor or HIPEC time, were not associated with increased morbidity. Only the number of procedures > 4 was significantly linked to an increase in morbidity.

Eight patients died in the postoperative period (1.66%). Four deaths were due to medical causes (PE, MI and liver failure), and the other deaths were due to causes directly related to the surgery (intestinal perforation, sepsis and lower GI bleeding). None of these deaths were directly related to the administration of HIPEC.

We found nine cases with complications that were linked exclusively to HIPEC (detected during the procedure) (1.9% of the total): six hyperglycaemia cases over 400 mg/dL, one allergy to oxaliplatin (anaphylactic shock), one significant metabolic acidosis and one case of hypercarbia (the only directly relatable with CO_2_ agitation). Seven cases were colon carcinomatosis (two appendiceal), and two cases were ovarian. The HIPEC duration was 30 min in 5 of the nine cases. The complications linked to HIPEC did not significantly increase the stay in the ICU.

Hyperglycaemia >400 mg/dL was related to carrier solution (5% dextrose) and was avoided, and in further procedures, carrier solution was switched to peritoneal dialysis fluid (Physioneal 35, with 1.36% glucose). With no known clinical significance of the difference, 5% dextrose maintains a concentration of oxaliplatin at levels that reach 101.2% at 60′ and 105.1% at 120′ of HIPEC, while peritoneal dialysis fluid levels slowly decrease to 91.7% at 60′ and 85.3% at 120′ of the original dosage, but avoids the serious hyperglycaemia and electrolyte disturbances caused by the former (5% dextrose). [36].

### 3.5. Survival Curves

**Figure 4:**
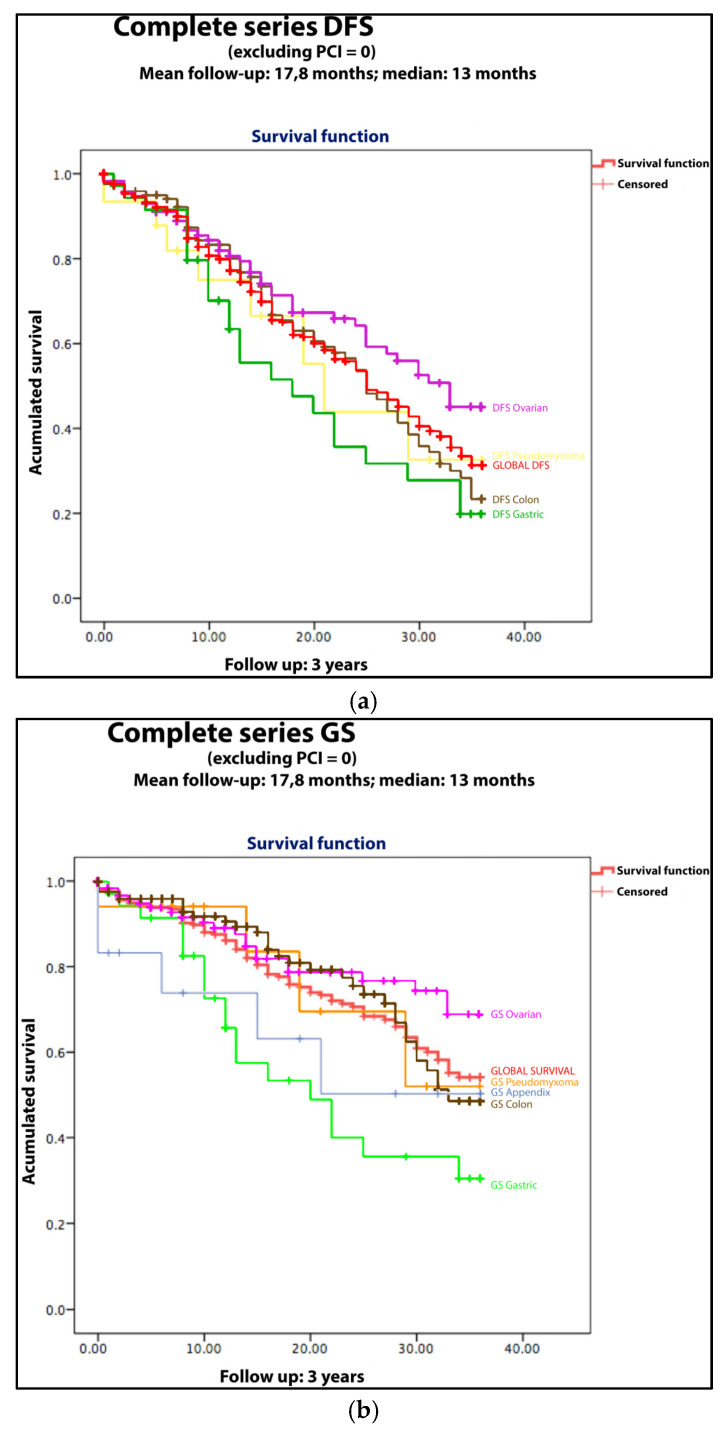
Survival curves by histology. (**a**) DFS: disease-free survival; ovarian (pink), global group (red), pseudomyxoma (orange) appendix (blue), colon (brown), gastric (green). (**b**) GS: global survival); ovarian (pink), pseudomyxoma (yellow), global group (red), colon (brown), gastric (green).

The OS of the series with a mean follow-up of 17.8 months was 86.1% and 54.1% after the first and third years, with DFS rates of 77.2% and 31.4%, respectively; a direct comparison with the main series can be seen in Table 4, Table 6, and Figure 4. The data by tumoral histology are detailed in Figure 2.

## 4. Discussion

Cytoreductive surgery in the treatment of peritoneal carcinomatosis is a useful tool in centers with experience and appropriate patient selection [37] to increase overall and disease-free survival [38,39]. The rate of complications in these procedures, which sometimes require excision of the peritoneum and the resection of affected organs for the macroscopic elimination of the tumor, is very similar to other highly complex surgeries [38].

The role of intraperitoneal chemotherapy as a theoretical complementary treatment to surgery for the eradication of the residual microscopic tumor has not been completely demonstrated in prospective trials [40,41], which may be because it has a much less standardized protocol than surgery [42]. Therefore, each group uses different treatment protocols with different chemotherapeutic agents, times, temperatures and methods of application without any evidence of which protocol produces better results [39,43]. Therefore, it is difficult to obtain global and valid conclusions. Intraperitoneal chemotherapy is also used in other scenarios, such as the prophylaxis treatment of peritoneal carcinomatosis in high-risk tumors [44] or the treatment of malignant ascites [45].

Intraperitoneal chemotherapy acts directly on local tumor cells via various mechanisms. The chemotherapeutic drugs selected are generally hydrophiles, with high molecular-weight molecules to prevent the drugs from passing through the peritoneal barrier. This characteristic minimizes their passage into the bloodstream, decreases their systemic toxicity and achieves much higher intraperitoneal concentrations than would be possible or safe with systemic chemotherapy [46]. The selected agents must have a fast, direct cytotoxic effect on the residual tumor, which must not be larger than 2.5 mm because the chemotherapeutic agents will not completely permeate the full thickness of larger tumors during the recirculation time.

Hyperthermia theoretically acts in three ways [47,48,49,50,51]: the first mechanism produces a direct thermal cytotoxic effect on the tumor cell; the second mechanism increases the cytotoxicity of the chemotherapeutic agents; and the third mechanism increases the ability of the chemotherapeutic agent to penetrate inside tumoral implants. Hyperthermia itself seems to play a significant role in the efficacy of intraperitoneal chemotherapy, as Yonemura et al. founded: “HIPEC at 42–43 °C had better results than lower temperatures or no HIPEC (only CRS)” [52], but the ideal temperature in a varied range of chemotherapeutic agents still remains controversial, because not all chemotherapeutic drugs reach their maximum efficacy or stability at the same temperature [49]. Some recent publications related high temperatures (>41.4 °C) to lower survival rates [14], and other studies related these findings to the synthesis of heat shock proteins (HSP) inside tumor cells, which ultimately protected the tumor cells (“thermotolerance”) by reducing the apoptosis generated by the chemotherapeutic drugs or selecting the subpopulations of tumor cells that were most resistant to the administered chemotherapy [50]. HSP therapies are being investigated to prevent their protective actions and as a marker for cytotoxic drugs [47].

Therefore, the ideal temperature is not well defined and will likely vary depending on the drugs used when we have more knowledge of their behavior at high temperatures. However, we must be able to monitor the temperature of the chemotherapy very well, modify it and keep it constant and homogeneous within the cavity for maximum efficacy in all areas and to avoid heat damage in areas of possible accumulation.

Another significant influence on the efficacy of intraperitoneal chemotherapy is the intraabdominal pressure of the fluid. Increasing the intraabdominal pressure increases the penetration of the medicine into the cell layers of the tumor implant by collapsing the capillaries that wash the chemotherapeutic agents in the peritoneum and increases the concentration and permanence of the agent in contact with the tumoral cells [45,53].

The most widely used method for the administration of perioperative chemotherapy is Hyperthermic IntraPEritoneal Chemotherapy (HIPEC) [22,54] because it unites the cytotoxic effect of chemotherapeutic agents with the effect of thermal shock on tumor cells. The classic method described by P. Sugarbaker is the open or “Coliseum” method, in which the chemotherapeutic agents are dissolved in a carrier solution, enter the abdominal cavity and are manually moved continuously to reach all areas of the peritoneum. However, the great difficulty of this method is maintaining a constant temperature throughout the entire abdomen. There are also safety concerns because of the direct and long-term contact of the surgeons with the chemotherapeutic drugs. A closed method was subsequently described to avoid possible exposure of the staff to the chemotherapeutic agents and maintain a more homogeneous temperature within the cavity and a higher intraabdominal pressure to help the penetration of chemotherapeutic agents into the tumor cells. The problem with this technique is the early formation of adhesions that hinder the ability to reach all areas of the peritoneum and the potential accumulation of heat or chemotherapeutic agents in some areas, which could lead to lesions or increased toxicity [22].

After several experimental trials in pigs [23] verified the safety of the technique, we started to use a new method for the administration of HIPEC in 2012, which was the closed technique with CO_2_ recirculation (Combat PRS^®^, Madrid, Spain).

Regarding efficacy, our results are very promising, with a mean overall survival near 50% at 3 years in a pathology where the published survival without treatment is 6 months (49% in colon cancer, almost 70% in ovarian cancer and 30% for tumors when carcinomatosis appears as gastric cancer).

The grade III/IV morbidity of our series was 9.96%, which is within the expected range for a surgery of this complexity and consistent with other groups. The mortality was also within acceptable margins and was 1.66% in our series [40].

Various studies compared the classic open and closed methods, but no groups demonstrated that one procedure was better than the other. Therefore, the best application method of HIPEC remains controversial [1,2].

In our experience, morbidity was lower by using the closed technique with CO_2_ in comparison with previous literature [1,2,3,4,5,6,7,8,9]; thus, it seems to be a safe option. As the different studies used different systems to classify adverse events, a direct comparison using a metanalysis review is unfortunately not possible [28].

Based on the morbidity/mortality data of the entire process and the analysis of the complications directly related to HIPEC, we found that the severe adverse events were related either to the chemotherapeutic drug itself (anaphylactic shock) or the carrier medium (hyperglycemia). Only one case presented a plausibly related complication with CO_2_ agitaton, hypercarbia, and survived.

During the procedures, no accidents were documented for spillage or contamination of the operating room staff with the chemotherapeutic agents. One advantage for the Combat PRS^®^ system is it is easy to mount and the cavity is closed and the gas is recirculated through the device, and thus the risk of inhaling any vapor created when heating the medicine is reduced to a minimum. Therefore, the procedure is also safe for health care staff when it is performed in compliance with the established protocol and security measures.

A main limitation of the study is the variability between centers because each center used a different treatment regimen with different chemotherapeutic drugs and times. These differences make it difficult for a comparison of concrete chemotherapy added or time of the technique. The variability in protocol as well as different length of follow-up in the included patients will require additional studies.

## 5. Conclusions

According to the experience of our multi-center group, the closed system with CO_2_ agitation seems to be a safe procedure for the application of HIPEC for the patient and health care staff. Only one patient suffered hypercarbia, which could be related directly to the CO_2_ agitation use. This new protocol showed similar survival as the previously published series. The application of HIPEC with CO_2_ recirculation using the Combat PRS^®^ device, thus, seems to be a safe and effective procedure that may be added to the therapeutic arsenal in the multimodal treatment of peritoneal carcinomatosis.

## Figures and Tables

**Table 1:** Participating hospitals, patients provided (yearly tumoral histology) and evolution over time of the number of procedures. (Pseud: pseudomyxoma. Perit: primary peritoneal. Mesot: peritoneal mesothelioma. Sarco: peritoneal sarcoma. Append: appendix. Panc: pancreas).

Series	Colon	Ovarian	Gastric	Pseud.	Perit.	Mesot.	Sarco.	Append.	Panc.	Other	Total
2011		3									3
2012		10									10
2013	5	14	1								20
2014	15	8	9			2		1			35
2015	28	14	10	2		1	1	4			60
2016	28	25	9	4		1		3		1	71
2017	55	20	8	11	1	2		5	1	1	104
2018	70	46	11	14	1	2	1		6	2	153
2019	9	9	1	1		2		1	2	1	26
Total	210	149	49	32	2	10	2	14	9	5	482
							Cumulative Series 2019				
Fuenlabrada University Hospital							101				
Cuidad Real University Hospital							79				
Principe de Asturias University Hospital							85				
Gran Canaria Insular Hospital							71				
Madrid Sanchinarro University Hospital							47				
University of Malaga Regional University Hospital							45				
Fundación Alcorcon University Hospital							18				
Elche General University Hospital							14				
Reina Sofia University Hospital							14				
FJD University Hospital							2				
Virgen de Arraixaca University Hospital							6				
TOTAL							482				

**Table 2:** Types of tumours.

Tumour	Total	%	Clinical PCI	Surgery PCI
Colon	210	43.6	4.6 (3.89–5.31)	6.37 (5.44–7.30)
Ovarian	149	30.9	8.58 (7.47–9.70)	9.68 (8.49–10.88)
Gastric	49	10.2	4.04 (2.12–5.96)	4.89 (2.81–6.98)
Appendix	14	2.9	8.64 (7.54–9.30)	10.33 (9.25–11.10)
Pseudomixoma	32	6.6	8.59 (7.20–9.70)	11.78 (10.50–12.36)
Mesothelioma	10	2.1	19.63 (17.25–20.50)	21.78 (18.30–22.45)
Pancreas	9	1.9		
Other	4	0.8		
Primary peritoneal	2	0.4		
Endometrium	1	0.2		
Sarcoma	2	0.4		
Total	482	100%		

**Table 3:** Inclusion criteria [24].

- Complete Cytoreduction (R0 resective surgery) - Age < 75 years - Functional Status According to WHO (ECOG) ≤ 2 - Presence of Peritoneal Carcinomatosis - Absence of Extra-Abdominal Metastasis - Absence of Hepatic Metastasis requiring a major or nonresectable hepatectomy - Liver, Kidney and Bone Marrow function within these parameters: Total Bilirubin ≤ 1.5 times the upper limit of normal (ULN) GOT/GPT ≤ 2.5 times ULN AP ≤ 3 times ULN Serum Creatinine ≤ 1.5 times NFS Neutrophils > 1.5 × 103 Hb > 10 g/dL Platelets > 100,000

**Table 4:** Comparison of morbidity/mortality and survival among various series related to the HIPEC technique used.

Authors	Technique	No.	Year	Tumour	Mortality (%)	Morbidity (%)	OS	DFS	Classification
Sugarbaker et al. [29]	Open	356	2006	AP	2	19	-	-	IV (proprietary base)
Elias et al. [30]	Open Closed	523	2010	CRC	3.3	31	1 y: 81% 3 y: 41% 5 y: 27%	1 y: 47% 3 y: 15% 5 y: 10%	CD: III/IV CTCAE
Goére [11] (PSOGI)	Open Closed	781	2017	Rare OC, Sarcomas, NT	2.9	41	1 y: 78% 3 y: 52% 5 y: 39%	1 y: 61% 3 y: 33% 5 y: 28%	CTCAE 4
Glehen et al. [31]	Closed	207	2003	OC, CRC, GC, PMP, PM, others	3.2	24.5	-	-	CD: III/IV
Kusamura et al. [32]	Closed	205	2006	OC, CRC, GC, PMP, PM, others	0.9	12	-	-	Bozzetti: 3–4
Levine et al. [33]	Closed	460	2007	OC, CRC, GC, PMP, PM, PS, others	4.8	43	3 y: 60%	-	Not described
Manzanedo et al. [15] (GECOP)	Open Closed PRS	88	2019	GC	3.4	31	1 y: 80% 3 y: 31%	1 y: 46% 3 y: 22%	CD (v2004): III/IV
Sanchez-Garcia et al. [34]	Closed PRS	21	2016	OC	4.76	38.1	-	-	CD: III/IV CTCAE 4
Cianci [35]	Closed PRS	17	2018	CRC, OC, AP, GC	0	38.1	-	-	CD: III/IV
Our group (Spain)	Closed PRS	482	2019	CRC, AP, GC, PMP, OC, others	1.66	9.96	1 y: 86% 3 y: 54%	1 y: 77% 3 y: 31%	CD (v2004): III/IV

GC: gastric cancer. CRC: colorectal cancer. AP: appendiceal cancer. OC: ovarian cancer. PMP: pseudomyxoma. PM: peritoneal mesothelioma. NT: neuroendocrine tumor. CTCAE: Common Terminology Criteria for Adverse Events. CD: Clavien-Dindo. GECOP: Spanish group of peritoneal oncologic surgery. Note: “Mortality” equals Clavien-Dindo V. 1 y: 1 year. 3 y: 3 year. 5 y: 5 year.

**Table 5:** Part **(a)** Relation between variables and increased morbidity; Part **(b)** Postoperative deaths (Clavien-Dindo V) (Decade 1 = 0–9 years; decade 2 = 10–19 years, and so on); Part **(c)** HIPEC specifically-related complications (CD II).

**(a)**									
**Complications CD III/IV**				**Statistic**			***p* Value**	**Risk**	**CI**
>4 procedures				Chi squared			0.035	1.928	(1.16–3.20)
Surgical PCI				Mann–Whitney U			0.154		
Age				Mann–Whitney U			0.888		
Type of primary tumour				Chi squared			0.387		
Medicine				Chi squared			0.103		
Sex				Chi squared			0.088		
HIPEC time				Mann–Whitney U			0.793		
**(b)**									
**Case ID**	**Age** **(Decade)**		**Histology**	**Postop Day**	**HIPEC Drugs**	**HIPEC Time**	**ICU Days**	**Cause of Death**	
HUCR11	7 th		Ovarian	8	Paclitaxel	60	7	Probable PE, CRA	
HUCR35	8 th		Ovarian	12	Paclitaxel	60	8	Intestinal perforation Peritonitis Multi-organ failure	
HUCR36	7 th		Ovarian	3	Paclitaxel	60	17	Intestinal perforation Intestinal ischemia Multi-organ failure	
HMS12	5 th		Colon	17	Oxaliplatin	60	79	Sepsis	
HMS34	8 th		Colon	20	Oxaliplatin	45	18	MI	
HUPA70	6 th		Pseudomyxoma	35	Mitomycin C + 5FU + Folinic	90	3	PE	
HRUM18	8 th		Colon	3	Oxaliplatin	30	8	Post-operative LGIB	
HUFLB	10 th		Colon		Oxaliplatin	30	8	Fatal and unexpected liver failure	
**(c)**									
**Code**	**Age** **(Decade)**	**T. 1º**	**>4 Proc**	**PCI _PRE-SURGICAL_**	**PCI _IN SURGERY_**	**HIPEC Drugs**	**Time**	**Complication**	**Days in ICU**
HUPA34	8 th	Colon	Yes	7	6	Oxaliplatin + Leucovorin + 5FU	10	Anaphylactic shock	7
HRUM12	4 th	Colon	Yes	30	30	Oxaliplatin	30	Hyperglycaemia	2
HRUM15	7 th	Colon	No	4	4	Oxaliplatin	30	Metabolic acidosis	2
HRUM18	7 th	Colon	No	3	3	Oxaliplatin	30	Hyperglycaemia	8
HRUM21	5 th	Ovary	No	2	3	Cisplatin + Doxorrubicin	90	Hyperglycaemia	2
HRUM22	7 th	Colon	No	6	23	Oxaliplatin	30	Hyperglycaemia	2
HRUM30	6 th	Colon	Yes	4	7	Mitomycin	60	Hyperglycaemia	2
HRUM40	6 th	Colon	No	2	3	Oxaliplatin	30	Hyperglycaemia	3
HGUE	5 th	Ovary	Yes	13	13	Paclitaxel	45	Hypercarbia	3

PE: pulmonary embolism. CRA: cardiorespiratory arrest. MI: acute myocardial infarction. LGIB: lower gastrointestinal bleeding.

**Table 6:** OS and DFS, by tumour histology, 1 and 3 years after HIPEC procedure.

Tumour Origin	Mean Follow-Up _(Months)_	OS _1 Year_	OS _3 Years_	DFS _1 Year_	DFS _3 Years_
Colon carcinomatosis	17.7	90.7%	48.7%	80.1%	23.4%
Appendiceal carcinomatosis	17.5	92.3%	64.6%	75.2%	51.6%
Ovarian carcinomatosis	18.8	89.1%	68.9%	80.8%	45.2%
Gastric carcinomatosis	17.3	65.8%	30.6%	63.5%	19.8%
Pseudomyxoma	14	84.2%	52.6%	76.3%	33.9%
Mesothelioma	16.2	50%	50%	50%	30%

## Data Availability

The data used to support the findings of the present study are available from the corresponding author upon request.

## Supplementary Materials

The following supporting information can be downloaded at: https://www.mdpi.com/article/10.3390/jcm11206152/s1, Spanish PRS collaborating group: Dr. Israel Manzanedo, on behalf of the Department of General and Digestive Surgery, Hospital Universitario Fuenlabrada, Madrid, Spain. Dra. Susana Sánchez García, on behalf of the Department of General and Digestive Surgery, Hospital Universitario of Ciudad Real. Dra. Laura González Sánchez, on behalf of the Department of General and Digestive Surgery Hospital Insular de Gran Canaria. Dr. Eduardo Díaz Reques, on behalf of the Department of General and Digestive Surgery, Hospital Universitario Madrid Sanchinarro. Dr. Alberto Titos García, on behalf of the Department of General and Digestive Surgery, Hospital Regional Universitario de Málaga. Dr. Manuel E. Marcello Fernández, on behalf of Hospital Universitario Fundación Alcorcón, Madrid, Spain. Dr. Ibán Caravaca García, on behalf of Hospital General Universitario de Elche, Alicante, Spain. Dr. Álvaro Arjona, on behalf of the Department of General and Digestive Surgery, Hospital Reina Sofía, Córdoba, Spain. Dr. Pedro Villarejo Campos, on behalf of Hospital Universitario Fundación Jiménez Díaz. Dr. Pedro Cascales Campos, on behalf of the Department of General and Digestive Surgery, Hospital Virgen de la Arrixaca, Murcia, Spain.
