# Supplementary material for: Hyperthermic Intraperitoneal Chemotherapy and Recirculation with CO2: A Safe Technique
Source: J Clin Med. 2022 Oct 19;11(20):6152. doi: 10.3390/jcm11206152 (PMC9605477; doi:10.3390/jcm11206152)
Supplement: Supplementary file 1 [file jcm-11-06152-s001.zip › jcm-1941454-supplementary.pdf]

Supplementary Materials 1: Spanish PRS collaborating group

Dr. Israel Manzanedo, on behalf of the Department of General and Digestive Surgery, Hospital Universitario Fuenlabrada, Madrid, Spain. Dra. Susana Sánchez García, on behalf of the Department of General and Digestive Surgery, Hospital Universitario of Ciudad Real. Dra. Laura González Sánchez, on behalf of the Department of General and Digestive Surgery Hospital Insular de Gran Canaria. Dr. Eduardo Díaz Reques, on behalf of the Department of General and Digestive Surgery, Hospital Universitario Madrid Sanchinarro. Dr. Alberto Titos García, on behalf of the Department of General and Digestive Surgery, Hospital Regional Universitario de Málaga. Dr. Manuel E. Marcello Fernández, on behalf of Hospital Universitario Fundación Alcorcón, Madrid, Spain. Dr. Ibán Caravaca García, on behalf of Hospital General Universitario de Elche, Alicante, Spain. Dr. Álvaro Arjona, on behalf of the Department of General and Digestive Surgery, Hospital Reina Sofía, Córdoba, Spain. Dr. Pedro Villarejo Campos, on behalf of Hospital Universitario Fundación Jiménez Díaz. Dr. Pedro Cascales Campos, on behalf of the Department of General and Digestive Surgery, Hospital Virgen de la Arrixaca, Murcia, Spain.
